# Pre-transplant heart disease etiology has a major effect on post-transplant survival of patients bridged with heartmate II assist device

**DOI:** 10.1186/1749-8090-8-S1-O155

**Published:** 2013-09-11

**Authors:** M Urban, J Pirk, O Szarszoi, I Netuka

**Affiliations:** 1 Department of Cardiac Surgery, Institute for Clinical and Experimental Medicine, Prague, Czech Republic

## Background

Left ventricular assist devices (LVAD) have become an established mode of treatment in bridging patients with advanced heart failure to cardiac transplantation. In certain groups of patients LVADs have proven their efficacy as a destination therapy. Increasing mismatch between the expanding pool of heart failure patients and decline in availability of donor organs raises an important question of prioritizing patients on the waiting list.

## Methods

This is a retrospective analysis of post-transplant results of patients bridged with HeartMate II. Patients were divided based on pre-transplant etiology into ischemic, non-ischemic and congenital group. One-year post-transplant survival was calculated using the Kaplan-Meier method and Log-rank test was used for comparison.

## Results

Between 2007 and 2013 60 patients were transplanted from HeartMate II at our institution. There were no differences in patients’ pre-transplant demographic and clinical variables. Donor characteristics as well as ischemic times were also comparable. Overall one-year post-transplant survival was 90±2%. Patient with non-ischemic etiology had 97±3% survival compared to 86±7% in patients with ischemic and 67±19% in patients with congenital heart disease (p = 0.043).

## Conclusion

Our data indicate that patients with congenital heart disease bridged to transplantation with HeartMate II have significantly reduced one-year post-transplant survival compared to patients with other cardiomyopathies. In the era of the current donor shortage, there is an increased pressure at judicious utilization of resources and surgical outcomes are increasingly scrutinized. Every effort should be made at identifying risk factors for diminished post-transplant survival. We believe that advancements in LVADs technical design associated with improved survival and quality of life make new generation devices a viable alternative as a definitive treatment option for a very high risk transplant patients.

